# Biomarkers in Chronic Fatigue Syndrome: Evaluation of Natural Killer Cell Function and Dipeptidyl Peptidase IV/CD26

**DOI:** 10.1371/journal.pone.0010817

**Published:** 2010-05-25

**Authors:** Mary A. Fletcher, Xiao R. Zeng, Kevin Maher, Silvina Levis, Barry Hurwitz, Michael Antoni, Gordon Broderick, Nancy G. Klimas

**Affiliations:** 1 Department of Medicine, University of Miami Miller School of Medicine, Miami, Florida, United States of America; 2 Miami Veterans Health Care Center, Miami, Florida, United States of America; 3 Department of Psychology, University of Miami, Coral Gables, Florida, United States of America; 4 Department of Medicine, University of Alberta, Edmonton, Alberta, Canada; New York University, United States of America

## Abstract

**Background:**

Chronic Fatigue Syndrome (CFS) studies from our laboratory and others described decreased natural killer cell cytotoxicity (NKCC) and elevated proportion of lymphocytes expressing the activation marker, dipeptidyl peptidase IV (DPPIV) also known as CD26. However, neither these assays nor other laboratory tests are widely accepted for the diagnosis or prognosis of CFS. This study sought to determine if NKCC or DPPIV/CD26 have diagnostic accuracy for CFS.

**Methods/Results:**

Subjects included female and male CFS cases and healthy controls. NK cell function was measured with a bioassay, using K562 cells and ^51^Cr release. Lymphocyte associated DPPIV/CD26 was assayed by qualitative and quantitative flow cytometry. Serum DPPIV/CD26 was measured by ELISA. Analysis by receiver operating characteristic (ROC) curve assessed biomarker potential. Cytotoxic function of NK cells for 176 CFS subjects was significantly lower than in the 230 controls. According to ROC analysis, NKCC was a good predictor of CFS status. There was no significant difference in NK cell counts between cases and controls. Percent CD2+ lymphocytes (T cells and NK cells) positive for DPPIV/C26 was elevated in CFS cases, but there was a decrease in the number of molecules (rMol) of DPPIV/C26 expressed on T cells and NK cells and a decrease in the soluble form of the enzyme in serum. Analyses by ROC curves indicated that all three measurements of DPPIV/CD26 demonstrated potential as biomarkers for CFS. None of the DPPIV/C26 assays were significantly correlated with NKCC.

**Conclusions:**

By ROC analysis, NKCC and three methods of measuring DPPIV/C26 examined in this study had potential as biomarkers for CFS. Of these, NKCC, %CD2+CD26+ lymphocytes and rMol CD26/CD2+ lymphocyte, required flow cytometry, fresh blood and access to a high complexity laboratory. Soluble DPPIV/C26 in serum is done with a standard ELISA assay, or with other soluble factors in a multiplex type of ELISA. Dipeptidyl peptidase IV on lymphocytes or in serum was not predictive of NKCC suggesting that these should be considered as non-redundant biomarkers. Abnormalities in DPPIV/CD26 and in NK cell function have particular relevance to the possible role of infection in the initiation and/or the persistence of CFS.

## Introduction

Chronic Fatigue Syndrome (CFS) is characterized by persistent and unexplained fatigue resulting in severe impairment in daily function and is defined by symptoms, disability, and exclusion of medical and psychiatric conditions that could explain the fatigue [1], [2]. Population-based studies estimated the prevalence of CFS at 0.23% to 0.41% [3], [4]. Costs to the US economy were estimated at $9 billion in lost productivity and up to $24 billion dollars in health care expenditures annually [5]–[7]. Complications and co-morbidity can be severe. For example, CFS was associated with chronic or episodic cardiovascular and autonomic dysfunction [8]. Recent results from our group demonstrated reduced stroke volume and cardiac output in more severely afflicted CFS patients [9]. Reports suggested increased risk of cancer as well as suicide [10], [11]. CFS affects all ethnic groups and socio-economic strata of society though at least 2 to 4 times as many women as men suffer from this illness [3], [12], [13]. Diagnosis using the case definition [1] requires the exclusion of any other medical explanation for these symptoms, yielding an inefficient, slow, error prone process. This is also costly because current clinical diagnosis typically involves tertiary care specialists.

Like many chronic illnesses CFS pathophysiology is complex and affects several of the body's main regulatory systems. There is a considerable literature describing immune dysfunction in CFS [14]–[16], although reviews of the immunology of CFS noted that universal agreement of immunological abnormalities had not been achieved, in no small part due to differences in methodologies, case definition and study quality [17], [18]. However, redundant reports support 1) reduced function of natural killer (NK) cells [14], [19] with deficiencies of perforin and granzymes in both NK cells and CD8 T cells [20]; 2) inflammation [21], [22]; 3) altered cytokine profiles [9], [10] with elevation of proinflammatory cytokines [11], [12] and Th2 (T helper cell type 2) polarization [11], [13]; and 4) chronic lymphocyte activation [14], [16].

Current research efforts are directed toward identifying an individual marker or combination of markers sufficiently associated with CFS to facilitate objective diagnosis and management of CFS. Previously we reported that CFS patients with poor NK function had more fatigue, less vigor, more daytime dysfunction, and more cognitive impairment. Those results provided preliminary evidence in support of using NKCC as subgroup marker for disease severity in CFS [23].

Present on the surface of many cells including lymphocytes, DPPIV/CD26 is a transmembrane glycoprotein and a serine peptidase that spits proline dipeptides from the N-terminus of polypeptides, including chemokines and neuropeptides. An enzymatically active soluble form is found in serum. We have observed an elevated proportion of lymphocytes expressing this activation marker in CFS patients as compared to controls [14].

No widely accepted laboratory tests are available for the diagnosis or prognosis of CFS. This study sought to determine the accuracy by which measurements of NKCC or DPPIV/CD26 distinguished between subjects with the clinically derived diagnosis of CFS and matched healthy controls.

## Methods

### Objectives

Prior work indicated defective NK cell function and a high percent of T cells and NK cells expressing the activation marker DPPIV/CD26 in CFS cases. The aim of this study was to determine the potential of NKCC and DPPIV/CD26 as biomarkers for CFS.

### Participants

Chronic fatigue syndrome patients (age 18 to 60, mean age 44; 83% female) were drawn from the University of Miami Miller School of Medicine CFS and Immunodeficiency Clinic after they were diagnosed with CFS using the CDC clinical diagnostic criteria [1], [2] (Table 1). All were participants in research studies (NIH, Chronic Fatigue and Immunodeficiency Syndrome Association (CFIDS) or University of Miami). Exclusion criteria included any active medical condition that could explain the presence of chronic fatigue, including diabetes, the current use of immunomodulatory or antibiotic medications, and a past or present psychiatric diagnosis of psychosis (e.g., schizophrenia), dementia, major depressive disorder with psychotic or melancholic features, bipolar disorder, anorexia or bulimia nervosa, or alcohol/substance abuse within two years of the onset of the fatigue or anytime thereafter. The CFS subjects were studied at 2 to 25 years after onset of symptoms, with an average onset of 10 years. Healthy controls (Table 1) (age 23–74, mean age 41, 86% female) were drawn from University of Miami, NIH or CFIDS funded studies. Each completed a medical and psychiatric history that included medications and alcohol/substance abuse. Those with active medical or psychiatric conditions, immunomodulating medications or alcohol/substance abuse were excluded.

**Table 1:** Natural killer cell cytotoxicity and dipeptidyl peptidase IV/CD26 in chronic fatigue syndrome cases^a^ compared to controls^b^.

*Variable*	*Number of CFS Cases*	*Median (25–75^th^ percentile)*	*Number of Healthy Controls*	*Median (25–75^th^ percentile)*	*p*
NKCC%	176	12 (8–21)	230	28 (20–37)	.000
% CD26+CD2+ Cells	75	61 (55–66)	100	52 (47–59)	.000
sCD26 in Serum (ng/ml)	73	489 (396–643)	122	671 (496–871)	.000
rMol CD26/CD2+ Cell	77	3625 (2844–4633)	102	4388 (3600–5388)	.001

**^a^>80% female, average age 48;**

**^b^>80% female, average age 47.**

### Description of Procedures or Investigations Undertaken

#### Blood Collection

Morning blood samples were collected. For lymphocyte function assays and flow cytometry, sodium heparin tubes were used. The samples were held at room temperature and delivered to the laboratory within 4 hours. For complete blood counts, the blood was collected into ethylene diamine tetra acetic acid and delivered to the laboratory within 4 hours. Serum was separated from blood clot within 4 hours of collection into red stopper tube and stored at −20°C until assayed.

#### Natural killer cell cytotoxicity

The bioassay for NKCC was performed using whole blood within 8 hours of collection in a chromium release assay as previously described [24]. The NK sensitive erythroleukemic K562 cell line was used as the target cell. The assay was done in triplicate at four target-to-effector cell ratios with 4-hour incubation. The % cytotoxicity at each target-to-effector ratio and number of CD3-CD56+ (NK) cells per unit of blood was used to express the results as % cytotoxicity at a target-to-effector cell ratio of 1∶1.

#### Determination of Lymphocyte Subsets and Assessment of Cell Surface Protein Concentrations by Quantitative Fluorescence

For the assessment of lymphocyte subsets, and the quantitative fluorescence intensity studies of cell surface antigen, a whole blood lysis method was used [25]. Whole blood samples were stained in 4 color combinations, with optimized (saturating) concentrations of antibodies, erythrocytes were lysed and the cell fixed with the Optilyse C reagent (Beckman-Coulter Corp., Hialeah, FL). Determination of lymphocyte, monocyte and granulocyte populations was determined using light scatter and back gating on fluorescence for the CD45 bright and CD14 negative population using a Beckman Coulter multiparameter flow cytometer. The isotype control was the reference for negative events. Spectral compensation was established daily. Quality control included optimization for lymphocyte recovery, purity of gate of analysis, lymphosum, and replicate determinations. Phycoerythrin (PE) labeled antibodies were used for quantitative fluorescence determinations and the median fluorescence intensity value was entered into a least squares linear regression equation derived from analysis of the QuantiBrite fluorescence intensity standards (Beckton Dickenson, San Jose, CA). This permitted conversion from fluorescence intensity values to median numbers of molecules PE bound per cell (relative numbers of molecules protein expressed per cell at saturating concentrations of antibody; rMol/cell). This technique allowed us to determine the relative (r) number of molecules (Mol) of CD26 on CD2+ lymphocytes (T cells and NK cells) (Figure S1).

#### Assay of Soluble CD26

Soluble CD26 in serum was assayed with an ELISA kit from Bender MedSystems (Vienna, Austria). This assay has a sensitivity of 7.26 ng/ml and precision of 4.6%.

#### Ethical issues

All subjects signed an informed consent approved by the University of Miami Institutional Review Board. Participants were English speaking with at least an 8^th^ grade education to ensure they were able to comprehend the informed consent as well as read and complete the questionnaires.

#### Statistical Methods

The nonparametric Mann-Whitney test was used to determine the magnitudes of between-group differences. The nonparametric Spearman test was used to determine correlations. Values of p<0.05 were considered statistically significant. The diagnostic accuracy of biomarkers was assessed in terms of true positive (sensitivity) versus true negative (1-specificity) using nonparametric receiver operating characteristics (ROC) analyses [26] available in the Statistical Package for Social Sciences (SPSS) software for Windows (SPSS Inc, Chicago, IL). The nonparametric ROC plot uses all of the data, makes no parametric assumptions and provides unbiased estimates of sensitivity and specificity, indicating the ability of a test to discriminate between two alternate states of health, in this case, CFS cases and healthy controls. The calculation of the area under the curve (AUC) provides a convenient single number. An AUC>0.5 indicates that the test shows no difference between the two groups while AUC = 1.0 is found if the test gives a perfect separation between groups. The coordinates of the curves (COC), which provide the entire spectrum of sensitivity/specificity pairs and a complete picture of test accuracy, are given in Supplementary Files for each ROC plot.

## Results

### Natural killer cell cytotoxicity

**Figure 1:**
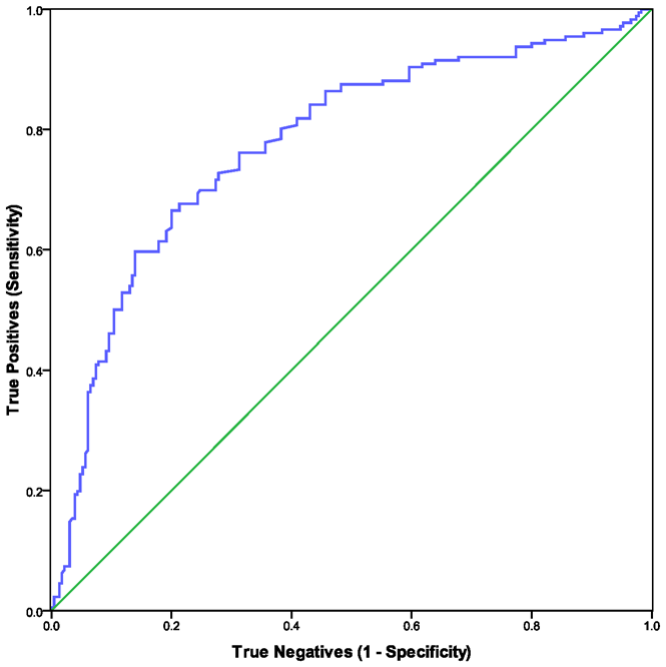
ROC analyses were used to evaluate NKCC as a predictor of CFS. The nonparametric ROC plot (blue curve) indicated the ability of NKCC to discriminate between CFS cases and healthy controls. Smaller values for NKCC were associated with CFS cases. The 45 degree line (green) indicates the theoretical plot of a test with no discrimination between CFS and controls.

The NKCC values were significantly lower in cases than controls (p<.000) (Table 1). Numbers of NK cells were not different between CFS and controls. The values for CD3-CD56+ lymphocytes/cumm (expressed as median (25^th^–75^th^ percentile) were: 176 (134–256) for CFS and 236 (151–336) for controls. According to the nonparametric ROC curve for 406 samples, as shown in Figure 1, NKCC was a good predictor of CFS status. Smaller values for NKCC indicated evidence for a positive actual state (CFS).The area under the curve (AOC) is shown in Table 2. The coordinates of the curve (COC) are given in Table S1.

**Table 2:** ROC curve analysis: Area Under the Curve (AUC) for natural killer cell cytotoxicity and dipeptidyl peptidase IV/CD26 in chronic fatigue syndrome cases compared to controls.

Variables	Area	Std. Error^a^	Asymptotic Sig.^b^	Asymptotic 95% Confidence Interval	
				Lower Bound	Upper Bound
NKCC%	.776	.024	.000	.729	.823
CD2+CD26+%	.746	.037	.000	.674	.818
sCD26 ng/ml	.732	.036	.000	.652	.794
rMolCD26/CD2+ cell	.650	.042	.001	.568	.733

**^a^Under the nonparametric assumption;**

**^b^Null hypothesis: true area = 0.5.**

### Dipeptidyl peptidase IV/CD26

**Figure 2:**
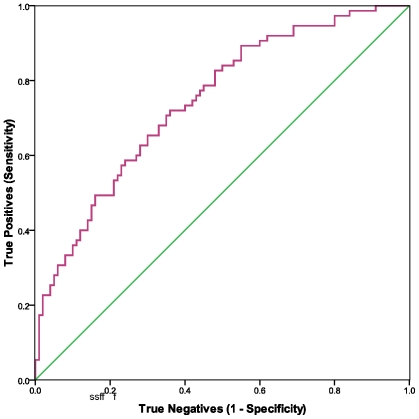
ROC analyses were used to evaluate %CD26+CD2+ lymphocytes as a predictor of CFS. The nonparametric ROC plot (purple curve) indicated the ability of %CD26+CD2+ lymphocytes to discriminate between CFS cases and healthy controls. Larger values for %CD26+CD2+ lymphocytes were associated with CFS cases. The 45 degree line (green) indicates the theoretical plot of a test with no discrimination between CFS and controls.

**Figure 3:**
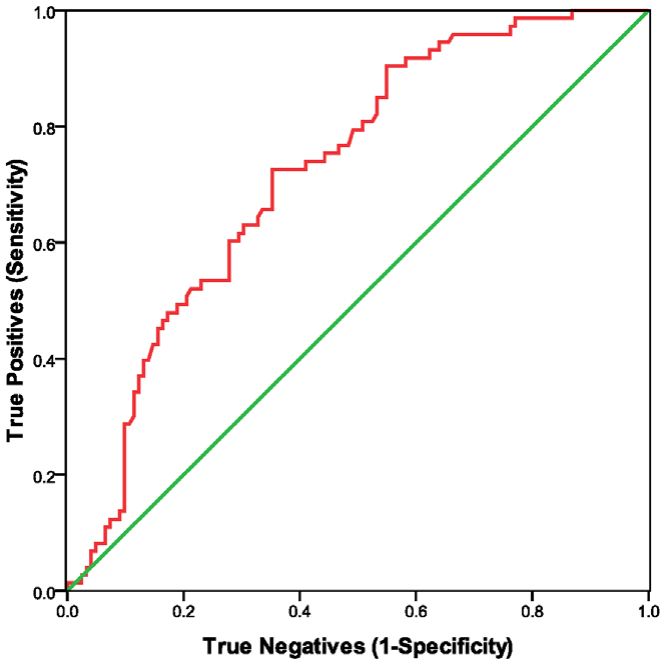
ROC analyses were used to evaluate serum dipeptidyl peptidase IV/CD26 as a predictor of CFS. The nonparametric ROC plot (red curve) indicated the ability of serum dipeptidyl peptidase IV/CD26 to discriminate between CFS cases and healthy controls. Smaller values were associated with CFS cases. The 45 degree line (green) indicates the theoretical plot of a test with no discrimination between CFS and controls.

**Figure 4:**
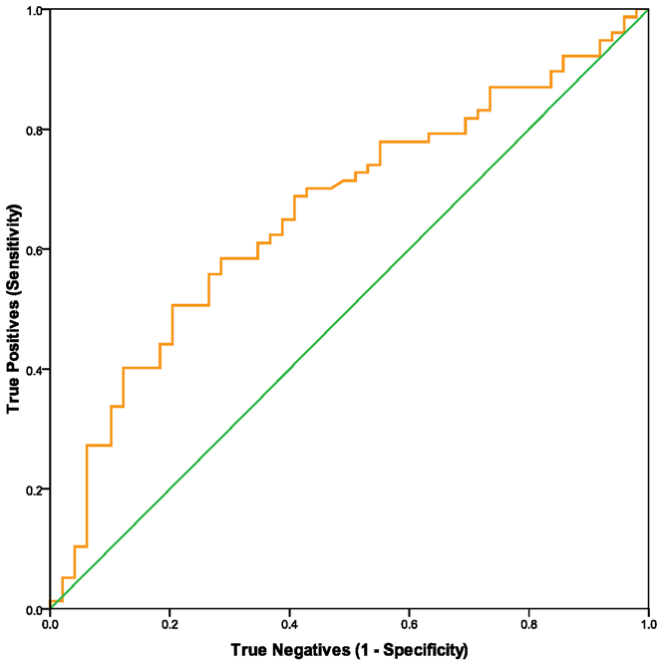
ROC analyses were used to evaluate relative number of molecules of dipeptidyl peptidase IV/CD26 on the surface of CD2+ lymphocytes as a predictor of CFS. The nonparametric ROC plot (orange curve) indicated the ability of number of molecules of dipeptidyl peptidase IV/CD26 on the surface of CD2+ lymphocytes to discriminate between CFS cases and healthy controls. Smaller values were associated with CFS cases. The 45 degree line (green) indicates the theoretical plot of a test with no discrimination between CFS and controls. cell at saturating concentrations of antibody; rMol/cell) is shown.

We measured this peptidase on cell surfaces and in serum in a subset of samples for which we had assayed NKCC. The results shown in Table 1, with CFS compared to controls, indicated an elevation of the percent of CD26+ CD2+ lymphocytes, but a decrease in the number of molecules of CD26 on T cells and NK cells and a decrease in the soluble form of CD26 in serum. ROC curve analyses and AUC, shown in Table 2 and Figures 2, 3, and 4 indicated that all three measures of CD26 have potential as biomarkers for CFS (see COCs in Tables S2, S3, and S4). The qualitative flow cytometry assay for proportion of CD26+CD2+ lymphocytes and the ELISA assay of sCD26 in serum were good predictors. The quantitative flow method for concentration of CD26 on CD2+ lymphocytes was less precise. Spearman analyses showed that none of the CD26 assays were significantly correlated with NKCC (data not shown).

## Discussion

Data from this and earlier studies gave credible support to diminished NKCC function in CFS. These effector cells of the innate immune system have an important role in antiviral, antibacterial, and antitumor immunity, but were deficient as measured by direct cytolysis of target cells, and as determined by measurement of intra cellular lytic proteins [14], [20]. In 60 to 80% of published samples, CFS presented with acute onset of illness, with systemic symptoms similar to influenza infection that did not subside [14]. The sudden onset, the symptoms of myalgia, arthralgia, sore throat and tender lymphadenopathy prompted a theory of infection induced illness [14], [27]. Published reports both support and deny associated microbial infections, reactivation of latent herpes virus infections and/or retrovirus infections in CFS [28]–[35]. Of interest is the finding by Glaser and colleagues that the adverse immunologic effects of persistent infections with Epstein Barr Virus (EBV) did not require viral DNA synthesis [36]. Some published work suggested the possibility of elevated risk for cancer in patients with CFS [10]–[11], though to date there has been no long term natural history study to accurately assess this risk.

Previously, we showed that the proportion of lymphocytes (NK cells and T cells) expressing CD26 is elevated in CFS cases [14]. In the present study, we found the density of DPPIV/CD26 on lymphocyte surfaces and the concentration of the enzyme in plasma is reduced in CFS subjects, compared to controls. We hypothesize that this reduction is due to chronic lymphocyte activation in CFS patients. The present study adds to the evidence of loss of innate immune function and chronic immune activation, resulting from the long term presence of antigenic stimulus, either self or foreign. Compared to healthy controls, chronic hepatitis C patients had significantly lower serum soluble CD26 levels [37]. In another study, acute, self-limiting infection with live influenza vaccine and chronic infection with persistent antigen, such as with cytomegalovirus (CMV), EBV or human immunodeficiency virus (HIV), was compared using multi-parameter flow cytometry and tetramer technology. These analyses identified a unique pattern of high density DPPIV/CD26 expression among influenza-specific CD8 T cells, but not among CD8 T cells specific for CMV, EBV (three different epitopes) or HIV [38]. These findings were interpreted as indicating that expression of CD26 (high) is characteristic of a memory cell, present in acute infection but not in chronic infection.

Dipeptidyl peptidase IV/CD26 cleaves N-terminal X-Pro dipeptides from peptides. The peptidase controls the *in vivo* half-life of the proinflammatory chemokine stromal cell-derived factor-1 (SDF-1). Mice deficient in DPPIV/CD26 exhibited increased levels of circulating active SDF-1, associated with increased numbers of SDF-1 receptor (CXCR4)-positive cells infiltrating arthritic joints [39]. In a clinical study, by the same researchers, plasma levels of DPPIV/CD26 from rheumatoid arthritis patients were significantly decreased when compared to those from osteoarthritis patients and inversely correlated with C-reactive protein levels. They postulated that decreased circulating soluble DPPIV/CD26 levels in arthritis may influence DPPIV/CD26-mediated regulation of the chemotactic SDF-1/CXCR4 axis. These patients have elevated number of T cells expressing DPPIV/CD26 and reduced DPPIV enzymatic activity and DPPIV/CD26 antigen in plasma compared to controls [39], [40].

Dipeptidyl peptidase IV/CD26 causes the degradation of glucagon-like peptide 1 (GLP-1), an incretin hormone [41]. Inhibitors of DPPIV/CD26 such as sitagliptin, which prevent the degradation of GLP-1 [42], are now marketed for the treatment of type 2 diabetes mellitus (T2DM). Considering that DPPIV/CD26 has a key role in immune regulation as a T cell activation molecule and in immune-mediated disorders, it is noteworthy that the effects of inhibition of DPPIV/CD26 on the immune system have not been extensively investigated. There are reports that infections were increased after sitagliptin treatment [43]. So far, only routine laboratory safety variables have been measured in published randomized controlled trials.

Administration of DPPIV/C26 inhibitors for the treatment of T2DM patients could influence immune function, including NKCC. A study of CD26 gene knockout mice concluded that DPPIV/C26 contributes to the regulation of development, maturation and migration of CD4 T, NK and NKT cells, cytokine secretion, T cell-dependent antibody production and immunoglobulin isotype switching of B cells [44]. An initial diagnosis of CFS would not be made in the patient with obvious T2DM. However, the frequency of development of T2DM after diagnosis of CFS is not known–nor is the effects of a DPPIV/C26 inhibitor in the CFS patient.

Duration of illness typically exceeds 10 years. Persistence may involve complex interaction of immune, autonomic and neuroendocrine regulation and remains poorly understood. It is important to recall that the associated chronic inflammation can have important consequences on energy metabolism by promoting insulin resistance [45]. This chronic inflammatory state would also support a concurrent low-grade Th1 response by inhibiting the protective effects of T regulatory cell subset via increased IL-6 expression. The decreased NKCC and the abnormal DPPIV/C26 manifestations in CFS would be compatible with the hypothesis that the immune system of affected individuals is biased towards a T- helper (Th) 2 type, or humoral immunity-oriented cytokine pattern.

The data obtained on NK cell function, immune activation and DPPIV/C26 on cell surfaces and in serum, are consistent with a viral etiology for CFS. The elevated proportion of activated CD4 and CD8 T cells and defective NKCC in CFS cases suggests that T cells are metabolically limited in performing their helper function. The abnormalities observed may have applications with other complex, chronic and poorly understood illnesses, including fibromyalgia, gulf war illness, rheumatologic disorders and multiple sclerosis—though the precise constellation of patterns observed with these biomarkers may differ in each. However, the specific panel that we have identified here are likely to be helpful as objective markers for diagnosing CFS, determining subgroups, following patients over time and as targets for therapeutic strategies. These indicators are parts of a complex and integrated system and their inter-dependency must be addressed [46]. Accordingly, we are currently engaged in mapping the network structure of neuroendocrine-immune interaction in CFS

### Limitations

Obvious limitations of this study are that each patient sample represents a single point in time. To address this, we are conducting a large longitudinal study to follow 150 subjects over 18 months. Samples are collected during times of relative symptom remission and exacerbation. Completion of the study will allow the correlation of CFS related symptoms with lymphocyte function and activation. Because CFS is a condition that affects women in disproportionate numbers, over eighty percent of the cases in the present study were female. The larger study will have sufficient power to allow a sub study of biomarker patterns in men with CFS.

### Conclusions

The predominance of evidence indicating that people with CFS have decreased function of NK cells and abnormal activation of T and NK cells was supported by this study. The purpose of the study was to determine usefulness of these measurements as biomarkers. By ROC analysis, NKCC and dipeptidyl peptidase/CD26 were identified as potential biomarkers for CFS through their demonstrated accuracy in discriminating CFS patients from healthy controls. Dipeptidyl peptidase/CD26 on lymphocytes or in serum was not correlated with NKCC, suggesting that these are non-redundant biomarkers. Current CFS treatments are directed at reducing symptom severity but no cure exists for this condition. The findings of this study give support to the concept that cause and/or the pathophysiology of CFS are related to infection. These findings may lead to therapeutic approaches. The specter of infectious disease further emphasizes the significance of this research to public health.

## Supporting Information

Figure S1 Illustration of technique used to convert fluorescence intensity values to median numbers of molecules PE bound per cell (relative numbers of molecules protein expressed per cell at saturating concentrations of antibody; rMol/cell).
(0.38 MB TIF)

Table S1 Coordinates of the Curve for NKCC.
(0.23 MB DOC)

Table S2 Coordinates of the ROC Curve for CD26+CD2+ Lymphocytes in CFS Compared to Controls.
(0.20 MB DOC)

Table S3 Coordinates of the ROC curve for sCD26.
(0.17 MB DOC)

Table S4 Coordinates of the curve for rMolCD26CD2+.
(0.28 MB DOC)
