# Supplementary figures and images for: Biomarkers in Chronic Fatigue Syndrome: Evaluation of Natural Killer Cell Function and Dipeptidyl Peptidase IV/CD26
Source: PLoS One. 2010 May 25;5(5):e10817. doi: 10.1371/journal.pone.0010817 (PMC2876037; doi:10.1371/journal.pone.0010817)

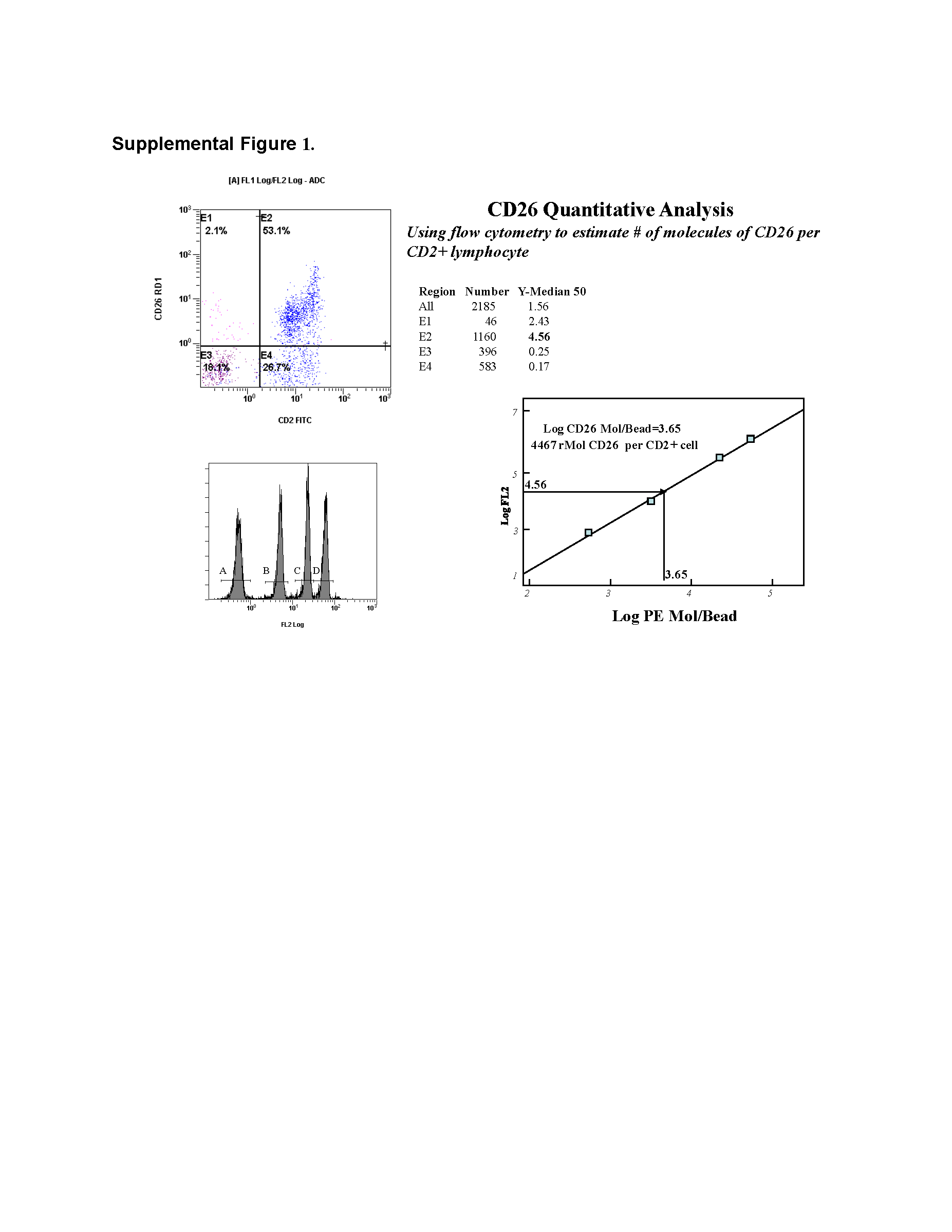

Supplement: Figure S1 — Illustration of technique used to convert fluorescence intensity values to median numbers of molecules PE bound per cell (relative numbers of molecules protein expressed per cell at saturating concentrations of antibody; rMol/cell). (0.38 MB TIF) [file pone.0010817.s001.tif]
