# Supplementary material for: Biomarkers in Chronic Fatigue Syndrome: Evaluation of Natural Killer Cell Function and Dipeptidyl Peptidase IV/CD26
Source: PLoS One. 2010 May 25;5(5):e10817. doi: 10.1371/journal.pone.0010817 (PMC2876037; doi:10.1371/journal.pone.0010817)
Supplement: Table S2 — Coordinates of the ROC Curve for CD26+CD2+ Lymphocytes in CFS Compared to Controls. (0.20 MB DOC) [file pone.0010817.s003.doc]

| **Supplemental Table S2: Coordinates of the ROC Curve for % CD26+CD2+ Lymphocytes in CFS Compared to Controls** | | |
| --- | --- | --- |
| Positive if Greater Than or Equal To^a^ | Sensitivity | 1 - Specificity |
| 27.83400 | 1.000 | 1.000 |
| 31.56700 | 1.000 | .990 |
| 34.99850 | 1.000 | .980 |
| 35.92400 | 1.000 | .970 |
| 36.17900 | 1.000 | .960 |
| 36.60350 | 1.000 | .950 |
| 37.20000 | 1.000 | .941 |
| 37.76950 | 1.000 | .931 |
| 38.56950 | 1.000 | .921 |
| 39.30000 | 1.000 | .911 |
| 39.93250 | .987 | .911 |
| 40.63250 | .987 | .901 |
| 41.17850 | .987 | .891 |
| 41.72850 | .987 | .881 |
| 42.13850 | .987 | .871 |
| 42.33950 | .987 | .861 |
| 42.55100 | .987 | .851 |
| 43.20000 | .987 | .842 |
| 44.08950 | .973 | .842 |
| 44.48950 | .973 | .832 |
| 44.68200 | .973 | .822 |
| 45.24250 | .973 | .812 |
| 45.81050 | .973 | .802 |
| 45.95000 | .960 | .802 |
| 46.00700 | .947 | .802 |
| 46.30700 | .947 | .792 |
| 46.61150 | .947 | .782 |
| 46.71350 | .947 | .772 |
| 46.85200 | .947 | .762 |
| 47.10000 | .947 | .752 |
| 47.51100 | .947 | .743 |
| 47.82550 | .947 | .733 |
| 48.00100 | .947 | .723 |
| 48.10700 | .947 | .713 |
| 48.14950 | .947 | .703 |
| 48.17900 | .947 | .693 |
| 48.25000 | .933 | .693 |
| 48.34950 | .920 | .693 |
| 48.40700 | .920 | .683 |
| 48.43350 | .920 | .673 |
| 48.47600 | .920 | .663 |
| 48.50900 | .920 | .653 |
| 48.52300 | .920 | .644 |
| 48.56900 | .920 | .634 |
| 48.65500 | .920 | .624 |
| 48.71750 | .907 | .624 |
| 48.74200 | .907 | .614 |
| 48.77450 | .907 | .604 |
| 48.83600 | .893 | .604 |
| 49.08600 | .893 | .594 |
| 49.31850 | .893 | .584 |
| 49.36850 | .893 | .574 |
| 49.42600 | .893 | .564 |
| 49.52600 | .893 | .554 |
| 49.87300 | .880 | .554 |
| 50.22300 | .880 | .545 |
| 50.40000 | .867 | .545 |
| 50.55100 | .853 | .545 |
| 50.72100 | .853 | .535 |
| 51.22000 | .853 | .525 |
| 51.61050 | .840 | .525 |
| 51.75300 | .840 | .515 |
| 52.09250 | .840 | .505 |
| 52.40000 | .840 | .495 |
| 52.59550 | .827 | .495 |
| 52.74500 | .827 | .485 |
| 52.89950 | .827 | .475 |
| 53.45000 | .813 | .475 |
| 54.00000 | .800 | .475 |
| 54.13000 | .787 | .475 |
| 54.28700 | .787 | .465 |
| 54.43000 | .787 | .455 |
| 54.52300 | .787 | .446 |
| 54.63500 | .773 | .446 |
| 54.68500 | .773 | .436 |
| 54.81750 | .760 | .436 |
| 54.96750 | .760 | .426 |
| 55.02250 | .747 | .426 |
| 55.07250 | .747 | .416 |
| 55.30000 | .733 | .416 |
| 55.60000 | .733 | .406 |
| 55.85000 | .733 | .396 |
| 56.05050 | .720 | .396 |
| 56.21300 | .720 | .386 |
| 56.36250 | .720 | .376 |
| 56.61800 | .720 | .366 |
| 56.96800 | .720 | .356 |
| 57.13300 | .707 | .356 |
| 57.18300 | .707 | .347 |
| 57.30000 | .693 | .347 |
| 57.48750 | .680 | .347 |
| 57.73150 | .680 | .337 |
| 57.89400 | .680 | .327 |
| 57.95000 | .667 | .327 |
| 58.05050 | .653 | .327 |
| 58.20100 | .653 | .317 |
| 58.32700 | .653 | .307 |
| 58.42650 | .653 | .297 |
| 58.65000 | .640 | .297 |
| 58.85000 | .627 | .297 |
| 59.10000 | .627 | .277 |
| 59.33800 | .600 | .277 |
| 59.43800 | .600 | .267 |
| 59.51150 | .587 | .267 |
| 59.63050 | .587 | .257 |
| 59.74650 | .587 | .248 |
| 59.77750 | .587 | .238 |
| 59.82300 | .573 | .238 |
| 59.87300 | .573 | .228 |
| 59.95200 | .547 | .228 |
| 60.15200 | .547 | .218 |
| 60.30650 | .533 | .218 |
| 60.35650 | .533 | .208 |
| 60.65000 | .520 | .208 |
| 60.95000 | .507 | .208 |
| 61.02300 | .493 | .208 |
| 61.11750 | .493 | .198 |
| 61.26500 | .493 | .188 |
| 61.47200 | .493 | .178 |
| 61.61250 | .493 | .168 |
| 61.71100 | .493 | .158 |
| 61.85000 | .480 | .158 |
| 61.95800 | .467 | .158 |
| 62.15800 | .467 | .149 |
| 62.49300 | .427 | .149 |
| 62.69300 | .427 | .139 |
| 62.90000 | .413 | .139 |
| 63.14850 | .400 | .139 |
| 63.22300 | .400 | .129 |
| 63.37450 | .400 | .119 |
| 63.59200 | .373 | .119 |
| 63.69200 | .373 | .109 |
| 63.75250 | .360 | .109 |
| 63.85250 | .360 | .099 |
| 64.05000 | .347 | .099 |
| 64.38750 | .333 | .099 |
| 64.68600 | .333 | .089 |
| 64.79850 | .333 | .079 |
| 65.00000 | .320 | .079 |
| 65.20700 | .307 | .079 |
| 65.25350 | .307 | .069 |
| 65.29650 | .307 | .059 |
| 65.35000 | .293 | .059 |
| 65.41400 | .280 | .059 |
| 65.56400 | .280 | .050 |
| 65.75000 | .267 | .050 |
| 66.09350 | .253 | .050 |
| 66.44350 | .253 | .040 |
| 66.53650 | .227 | .040 |
| 66.74900 | .227 | .030 |
| 67.61250 | .227 | .020 |
| 68.35000 | .213 | .020 |
| 68.90000 | .200 | .020 |
| 70.00000 | .187 | .020 |
| 70.68750 | .173 | .020 |
| 70.88750 | .173 | .010 |
| 71.35000 | .160 | .010 |
| 72.65000 | .147 | .010 |
| 73.65000 | .133 | .010 |
| 73.95000 | .107 | .010 |
| 74.25000 | .093 | .010 |
| 74.95000 | .080 | .010 |
| 75.80000 | .067 | .010 |
| 76.39200 | .053 | .010 |
| 76.89200 | .053 | .000 |
| 77.75000 | .040 | .000 |
| 78.75000 | .027 | .000 |
| 82.60000 | .013 | .000 |
| 87.20000 | .000 | .000 |
| a. The smallest cutoff value is the minimum observed test value minus 1, and the largest cutoff value is the maximum observed test value plus 1. All the other cutoff values are the averages of two consecutive ordered observed test values. | | |
