# Supplementary material for: Biomarkers in Chronic Fatigue Syndrome: Evaluation of Natural Killer Cell Function and Dipeptidyl Peptidase IV/CD26
Source: PLoS One. 2010 May 25;5(5):e10817. doi: 10.1371/journal.pone.0010817 (PMC2876037; doi:10.1371/journal.pone.0010817)
Supplement: Table S3 — Coordinates of the ROC curve for sCD26. (0.17 MB DOC) [file pone.0010817.s004.doc]

| **Supplemental Table S3: Coordinates of the ROC Curve for Serum soluble CD26 in CFS Compared to Controls** | | |
| --- | --- | --- |
|  | | |
| Positive if Less Tlhan or Equal To^a^ | Sensitivity | 1 - Specificity |
| 121.0000 | .000 | .000 |
| 129.0000 | .014 | .000 |
| 178.0000 | .014 | .008 |
| 229.5000 | .014 | .016 |
| 245.0000 | .014 | .025 |
| 252.0000 | .027 | .025 |
| 255.5000 | .027 | .033 |
| 268.5000 | .041 | .033 |
| 285.0000 | .041 | .041 |
| 296.5000 | .055 | .041 |
| 303.5000 | .068 | .041 |
| 311.0000 | .068 | .049 |
| 327.9600 | .082 | .049 |
| 340.4600 | .082 | .057 |
| 343.0000 | .082 | .066 |
| 344.5000 | .096 | .066 |
| 345.5000 | .110 | .066 |
| 346.5000 | .110 | .074 |
| 351.5000 | .123 | .074 |
| 357.0250 | .123 | .082 |
| 360.5250 | .123 | .090 |
| 365.5000 | .137 | .090 |
| 370.5000 | .137 | .098 |
| 374.0000 | .164 | .098 |
| 382.0000 | .192 | .098 |
| 391.0000 | .205 | .098 |
| 394.0000 | .233 | .098 |
| 396.0000 | .247 | .098 |
| 400.0000 | .260 | .098 |
| 403.5000 | .274 | .098 |
| 405.0000 | .288 | .098 |
| 412.0000 | .288 | .107 |
| 419.5000 | .301 | .115 |
| 423.5000 | .315 | .115 |
| 426.5400 | .342 | .115 |
| 430.0400 | .342 | .123 |
| 434.5000 | .356 | .123 |
| 439.2650 | .370 | .123 |
| 443.2650 | .370 | .131 |
| 444.5000 | .384 | .131 |
| 449.9200 | .397 | .131 |
| 456.4200 | .397 | .139 |
| 459.0000 | .425 | .148 |
| 462.5000 | .425 | .156 |
| 465.5000 | .438 | .156 |
| 466.5450 | .452 | .156 |
| 470.5450 | .452 | .164 |
| 475.5000 | .466 | .164 |
| 477.5000 | .466 | .172 |
| 478.6400 | .479 | .172 |
| 479.6400 | .479 | .189 |
| 481.0000 | .493 | .189 |
| 485.5000 | .493 | .205 |
| 490.0000 | .507 | .205 |
| 491.5000 | .521 | .213 |
| 492.5000 | .521 | .230 |
| 494.5000 | .534 | .230 |
| 498.2550 | .534 | .254 |
| 501.2550 | .534 | .262 |
| 503.0000 | .534 | .270 |
| 508.0000 | .534 | .279 |
| 513.0000 | .548 | .279 |
| 516.5000 | .562 | .279 |
| 522.0000 | .575 | .279 |
| 530.0000 | .589 | .279 |
| 538.0000 | .603 | .279 |
| 541.7450 | .603 | .287 |
| 543.7450 | .603 | .295 |
| 548.0000 | .616 | .295 |
| 553.0000 | .616 | .303 |
| 556.0000 | .630 | .303 |
| 560.0000 | .630 | .311 |
| 563.1300 | .630 | .320 |
| 564.1300 | .630 | .328 |
| 569.5000 | .644 | .328 |
| 582.0000 | .658 | .336 |
| 591.0000 | .658 | .352 |
| 595.0000 | .671 | .352 |
| 604.5000 | .685 | .352 |
| 613.5000 | .712 | .352 |
| 616.5000 | .726 | .352 |
| 617.5000 | .726 | .361 |
| 618.5000 | .726 | .369 |
| 620.0000 | .726 | .377 |
| 622.5000 | .726 | .385 |
| 624.5000 | .726 | .393 |
| 626.0000 | .726 | .402 |
| 629.0000 | .726 | .410 |
| 631.5000 | .740 | .410 |
| 634.0000 | .740 | .426 |
| 636.1400 | .740 | .434 |
| 636.6400 | .740 | .443 |
| 638.5000 | .753 | .443 |
| 642.4600 | .753 | .451 |
| 645.4600 | .753 | .459 |
| 648.0000 | .753 | .467 |
| 650.3350 | .767 | .467 |
| 653.8350 | .767 | .475 |
| 662.0000 | .767 | .484 |
| 668.0000 | .795 | .492 |
| 671.0000 | .795 | .500 |
| 675.5000 | .795 | .508 |
| 680.0500 | .808 | .508 |
| 684.5500 | .808 | .516 |
| 689.0000 | .808 | .525 |
| 692.0000 | .822 | .533 |
| 693.5000 | .836 | .533 |
| 705.0000 | .849 | .533 |
| 716.5000 | .849 | .541 |
| 717.5000 | .849 | .549 |
| 718.5000 | .877 | .549 |
| 720.0000 | .890 | .549 |
| 723.0000 | .904 | .549 |
| 731.0000 | .904 | .566 |
| 737.5000 | .904 | .574 |
| 741.5000 | .904 | .582 |
| 746.0000 | .918 | .582 |
| 747.5000 | .918 | .590 |
| 750.0000 | .918 | .598 |
| 756.0000 | .918 | .607 |
| 761.0000 | .918 | .615 |
| 764.5000 | .918 | .623 |
| 768.0000 | .932 | .623 |
| 771.0000 | .932 | .631 |
| 779.0000 | .932 | .639 |
| 786.5000 | .945 | .639 |
| 789.0000 | .945 | .648 |
| 794.5000 | .945 | .656 |
| 800.0000 | .959 | .664 |
| 804.2235 | .959 | .672 |
| 809.7235 | .959 | .680 |
| 822.5000 | .959 | .689 |
| 834.0000 | .959 | .697 |
| 840.0000 | .959 | .705 |
| 845.5000 | .959 | .721 |
| 855.5000 | .959 | .730 |
| 868.0000 | .959 | .738 |
| 881.5000 | .959 | .754 |
| 892.5000 | .959 | .762 |
| 895.0000 | .973 | .762 |
| 900.5000 | .973 | .770 |
| 906.0000 | .986 | .770 |
| 917.0000 | .986 | .779 |
| 942.0000 | .986 | .787 |
| 961.5000 | .986 | .795 |
| 966.0000 | .986 | .803 |
| 979.0000 | .986 | .811 |
| 999.0000 | .986 | .820 |
| 1012.0000 | .986 | .828 |
| 1019.5000 | .986 | .844 |
| 1029.5000 | .986 | .852 |
| 1041.5000 | .986 | .861 |
| 1051.0000 | .986 | .869 |
| 1059.0000 | 1.000 | .869 |
| 1066.5000 | 1.000 | .877 |
| 1089.0000 | 1.000 | .885 |
| 1112.2150 | 1.000 | .893 |
| 1146.2150 | 1.000 | .902 |
| 1176.0000 | 1.000 | .910 |
| 1180.0000 | 1.000 | .918 |
| 1185.0000 | 1.000 | .926 |
| 1194.5000 | 1.000 | .934 |
| 1231.0000 | 1.000 | .951 |
| 1309.0000 | 1.000 | .959 |
| 1389.0000 | 1.000 | .967 |
| 1452.1100 | 1.000 | .975 |
| 1487.6100 | 1.000 | .984 |
| 1777.5000 | 1.000 | .992 |
| 2065.0000 | 1.000 | 1.000 |
| The test result variable(s): sCD26plasma,T1 has at least one tie between the positive actual state group and the negative actual state group. | | |
| a. The smallest cutoff value is the minimum observed test value minus 1, and the largest cutoff value is the maximum observed test value plus 1. All the other cutoff values are the averages of two consecutive ordered observed test values. | | |
